# Silibinin Attenuates Silica Dioxide Nanoparticles-Induced Inflammation by Suppressing TXNIP/MAPKs/AP-1 Signaling

**DOI:** 10.3390/cells9030678

**Published:** 2020-03-10

**Authors:** Je-Oh Lim, Na-Rae Shin, Yun-Soo Seo, Hyeon-Hwa Nam, Je-Won Ko, Tae-Yang Jung, Se-Jin Lee, Ha-Jung Kim, Young-Kwon Cho, Jong-Choon Kim, In-Chul Lee, Joong-Sun Kim, In-Sik Shin

**Affiliations:** 1 College of Veterinary Medicine (BK21 Plus Project Team), Chonnam National University, 77 Yongbong-ro, Buk-gu, Gwangju 61186, Korea; dvmljo@gmail.com (J.-O.L.); rheoda@gmail.com (J.-W.K.); jupiterriot@naver.com (T.-Y.J.); xhdhksdl123@naver.com (S.-J.L.); kimhj614@jnu.ac.kr (H.-J.K.); toxkim@jnu.ac.kr (J.-C.K.); 2 Research Institute of Radiation & Medical Science, Korea Institute of Radiation & Medical Sciences, Seoul 01812, Korea; tlsskfo87022@gmail.com; 3 Herbal Medicine Resources Research Center, Korea Institute of Oriental Medicine, Geonjae-ro 177, Naju-si, Jeollanam-do 58245, Korea; sys0109@kiom.re.kr (Y.-S.S.); hhnam@kiom.re.kr (H.-H.N.); 4 College of Health Sciences, Cheongju University, 298 Daesung-ro, Sangdang-gu, Cheongju-si, Chungbuk 28503, Korea; petmen@hanmail.net; 5 Functional Biomaterial Research Center, Jeonbuk Branch, Korea Research Institute of Bioscience and Biotechnology, 181 Ipsin-gil, Jeongeup-si, Jeonbuk 56212, Korea; leeic@kribb.re.kr

**Keywords:** silica dioxide nanoparticle, silibinin, airway inflammation, thioredoxin-interacting protein, mitogen-activated protein kinase, activator factor-1

## Abstract

Silica dioxide nanoparticles (SiONPs) have been applied to several fields, such as drug delivery and gene therapy. However, SiONPs are a constituent of fine dust and can induce excessive inflammatory responses in the lungs via the airways. Silibinin, a major component of silymarin, has been known for its anti-oxidant and anti-inflammatory effects. In the present study, we explored the protective effects of silibinin against SiONPs-induced airway inflammation and explored its underlying mechanism of action, focusing on thioredoxin-interacting protein (TXNIP)/mitogen-activated protein kinases (MAPKs) in vitro and in vivo. In SiONPs-stimulated NCI-H292 airway epithelial cells, silibinin treatment effectively suppressed the elevation of the mRNA expression of tumor necrosis factor-α (TNF-α), interleukin (IL)-6, and IL-1β, which was accompanied by the reduction in the expression of TXNIP, MAPKs, and activator protein-1 (AP-1). In SiONPs-treated mice, silibinin administration inhibited the increase in inflammatory cell counts and proinflammatory mediators, and it alleviated airway inflammation by SiONPs exposure. In addition, silibinin administration effectively suppressed the elevation of TXNIP/MAPKs/AP-1 signaling by SiONPs exposure. Taken together, silibinin effectively inhibited SiONPs-induced inflammatory responses, and this effect was closely related to the inhibition of TXNIP/MAPK/AP-1 signaling. These results suggested that silibinin might be useful for reducing pulmonary inflammation induced by SiONPs.

## 1. Introduction

Silica dioxide nanoparticles (SiONPs) are nanomaterials considered important in modern society because they offer various properties and applications, particularly in the field of biology, biotechnology, and medicine [1]. However, as SiONPs are not present in nature and are artificially produced, they become components of fine dust and can be a potential hazard to human health [2]. In particular, the lungs are the main target organ of SiONPs [3,4], and exposure to SiONPs is associated with pulmonary tuberculosis, chronic obstructive pulmonary disease, emphysema, silicosis, and lung cancer [5,6,7,8]. Moreover, SiONPs induce pathophysiological alterations in various cell types, including endothelial cells, lung cancer cells, and macrophages, and these alterations are mediated by reactive oxygen species (ROS), inflammation, apoptosis, and autophagy [9,10,11,12,13]. Owing to the recent increase in the use of SiONPs, their potential health risk has been increasing.

The immune system plays a protective role in our body; it responds to tissue injury, infection, and harmful pathogens that enter the body [14]. However, excessive inflammatory responses can lead to tissue damage, organ failure, and ultimately death [15]. Activation of mitogen-activated protein kinases (MAPKs) signaling is recognized to be mediated by many stimuli and is associated with the induction of cell death and inflammation [16]. In recent studies, exposure to SiONPs induces an inflammatory response in human lung cells, and this effect is associated with the elevation of the MAPKs signaling pathway [17,18]. In addition, thioredoxin-interacting protein (TXNIP) plays a role as an oxidative stress activator by decreasing the antioxidant activity of thioredoxin (TRX) [19]. A previous study has shown that the downregulation of TXNIP ameliorates inflammatory responses by inhibiting the MAPKs pathway in mice [20]. TXNIP alleviates inflammatory responses by suppressing p38 MAPK signaling in lipopolysaccharide (LPS)-stimulated macrophages [21]. Therefore, suppression of the TXNIP/MAPKs pathway can be a strategy for controlling SiONP-induced inflammation.

Silibinin, a natural polyphenolic flavonoid, is the main component of silymarin, which is extracted from milk thistle [22]. Silibinin has anti-oxidant, anti-cancer, and anti-inflammatory properties [23]. A recent study has shown that silibinin attenuates pulmonary inflammation in cigarette smoke-induced chronic obstructive pulmonary disease mouse models by suppressing the extracellular signal-regulated protein kinases 1 and 2 (ERK1/2)/specificity the protein 1 pathway [24]. Silibinin also inhibits cytokine-induced activation of activator protein-1 (AP-1), c-Jun N-terminal kinase (JNK), ERK1/2, and p38 in human lung carcinoma cells [25]. However, it is unknown whether silibinin exerts a pulmo-protective anti-inflammatory mechanism by regulating TXNIP in the lungs.

Therefore, we studied the effects of silibinin on airway inflammation induced by SiONPs exposure in vitro and in vivo. Furthermore, the action mechanism of silibinin was investigated with a focus on the TXNIP/MAPKs/AP-1 pathway.

## 2. Materials and Methods

### 2.1. Nanoparticles

The particle size of SiONPs (Sigma–Aldrich, St. Louis, MO, USA) is <5–15 nm, according to manufacturer’s information. SiONPs were dissolved in phosphate-buffered saline (PBS) and sonicated for 3 min before administration.

### 2.2. Cell Culture

The NCI-H292 cells, as human airway epithelial cell line (American Type Culture Collection, Manassas, VA, USA) were maintained at 37 °C with 5% CO_2_ in RPMI 1640 medium with fetal bovine serum (FBS, 10%), penicillin (100 U/mL), streptomycin (100 μg/mL), and HEPES (25 mM). Fresh media were exchange per every 2–3 days. The cells were starved in the medium with 1% FBS for 5 h before use.

### 2.3. Measurement of Proinflammatory Cytokines mRNA Expression in NCI-H292 Cells

Cells were seeded into six-well plates (4 × 10^5^ cells/well) for 24 h, and then treated with silibinin (Sigma–Aldrich) at 2.5, 5, 10, and 20 μg/mL based on the result of cell survival and TXNIP expression at basal condition (Supplementary Figures S1 and S2, respectively). The control and other treatments contained a 1:1000 dilution of dimethyl sulfoxide (DMSO). After incubation for 12 h, the cells were treated with SiONPs at 12.5 μg/mL for 6 h based on the preliminary experiment (Supplementary Figure S3). To perform real-time reverse-transcription polymerase chain reaction (qRT-PCR), total RNA was isolated using TrizolTM reagent (Invitrogen, Carlsbad, CA, USA) and then reverse-transcribed using a cDNA kit (Qiagen, Hilden, Germany). qRT-PCR experiments were performed using specific forward and reverse primers (TNF-α: forward, 5′-CAA AGT AGA CCT GCC CAG AC-3′, reverse, 5′-GAC CTC TCT CTA ATC AGC CC-3′; IL-6: forward, 5′- ATG CAA TAA CCA CCC CTG AC-5′, reverse, 5′- ATC TGA GGT GCC CAT GCT AC-3′; IL-1β: forward, 5′- AGC CAG GAC AGT CAG CTC TC-3′, reverse, 5′- ACT TCT TGC CCC CTT TGA AT-3′; GAPDH: forward, 5′-CAA AAG GGT CAT CTC TG-3′, reverse, 5′-CCT GCT TCA CCA CCT TCT TG-3′). We performed additional experiments using TXNIP small interfering RNA (siRNA) to investigate the role of TXNIP in the effects of silibinin on SiONPs-induced NCI-H292 cells. The siRNA for TXNIP (4392420) and scrambled siRNA (4390843) were purchased from Ambion (Waltham, MA, USA). Each siRNA (20 nM) was transfected into NCI-H292 cells using Lipofectamine^TM^ RNAiMAX reagent (Invitrogen, Waltham, MA, USA) following the forward transfection method as instructed by the manufacturer.

### 2.4. Animals

Specific pathogen-free female BALB/c mice (6 weeks old, Samtako Co., Osan, Korea) were used after 1 week of quarantine and acclimatization. The mice were maintained at normal conditions (temperature: 23 ± 2 °C, a relative humidity: 50 ± 5%, artificial lighting: 08:00–20:00 and air change: 13–18 per hour) and were provided a standard rodent diet and water ad libitum. The animal experimental procedures were performed according to the NIH Guidelines for the Care and Use of Laboratory Animals and were approved by the Institutional Animal Care and Use Committee of Chonnam National University.

### 2.5. Experimental Procedure for Animal Experiments

The mice were divided into one normal control (NC) group and four experimental groups, and each group consisted of six female mice. The experimental groups were divided into four groups as follows: SiONP group (SiONP instillation only), dexamethasone (DEX) group (SiONP instillation and 2 mg/kg DEX administration), and Sili 20 and 40 groups (SiONPs instillation and silibinin administration at 20 and 40 mg/kg, respectively). DEX and silibinin were daily treated by oral gavage to the mice for 2 weeks. SiONPs were administered to the mice at 20 mg/kg in 50 µL of PBS via intranasal instillation on days 1, 7, and 13 under slight anesthesia using Alfaxan (Alfaxalone^®^; Jurox Pty Ltd., Hunter Valley, Australia). The NC group were administered with 50 µL of PBS via intranasal instillation. A tracheostomy was performed under anesthesia at 48 h after the final instillation using Alfaxan. The procedure to collect bronchoalveolar lavage fluid (BALF) was performed as previously described [18]. Differential cell counts of BALF were determined using Diff-Quik^®^ reagent (IMEB Inc., San Marcos, CA, USA).

### 2.6. Measurements of Proinflammatory Cytokines in BALF

The IL-1β, IL-6, and TNF-α were determined by commercial enzyme-linked immunosorbent assay (ELISA) kits (BD Biosciences, San Jose, CA, USA). The absorbance was measured at 450 nm using a spectrophotometer (Bio-Rad Laboratories, Hercules, CA, USA).

### 2.7. Histology and Immunohistochemistry (IHC)

Lung tissues were fixed (4% (*v*/*v*) paraformaldehyde), embedded (paraffin), sectioned (thickness of 4 μm), and then stained with hematoxylin and eosin (Sigma–Aldrich) to estimate the status of inflammation. The procedure of IHC was the same as previously described [26]. Primary antibodies were used: TXNIP (1:200 dilution; Novus Biologicals, Littleton, CO, USA) and AP-1 (1:200 dilution; Abcam, Cambridge, UK) antibodies. Airway inflammation and protein expression were quantitatively measured using an image analyzer (IMT i-Solution software, Vancouver, BC, Canada).

### 2.8. Immunoblotting for Measuring Inflammatory Proteins in NCI-H292 Cells and Lung Tissues

Immunoblotting was the same as previously described [26]. The following primary antibodies were used: TXNIP (Novus Biologicals), phospho-ERK1/2 (p-ERK; Cell Signaling, Denver, MA, USA), total ERK (t-ERK; Cell Signaling), phospho-JNK (p-JNK; Cell Signaling), total JNK (t-JNK; Cell Signaling), phospho-p38 (p-p38; Cell Signaling), total p38 (t-p38; Cell Signaling), AP-1 (Abcam), and β-actin (Cell Signaling). The relative protein expression was determined using ChemiDoc (Bio-Rad Laboratories).

### 2.9. Statistical Analysis

Data were shown as means ± standard deviation (SD). Statistical significance was determined by analysis of variance followed by Dunnett’s test. *p*-values less than 0.05 were considered as statistical significance.

## 3. Results

### 3.1. Effects of Silibinin on the mRNA Expression of Proinflammatory Cytokines in NCI-H292 Cells Stimulated with SiONPs

**Figure 1:**
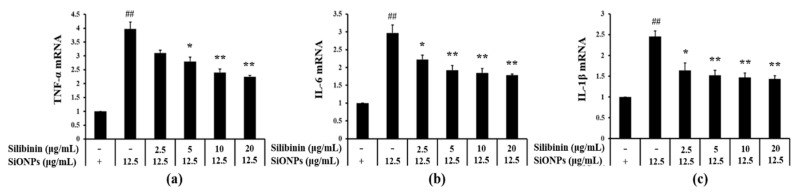
Effects of silibinin treatment on the mRNA expression of proinflammatory cytokines in silica dioxide nanoparticles (SiONPs)-induced NCI-H292 cells as assessed by real-time reverse-transcription polymerase chain reaction (qRT-PCR.) (**a**) mRNA expression of tumor necrosis factor-α (TNF-α). (**b**) mRNA expression of interleukin (IL)-6. (**c**) mRNA expression of IL-1β. Data are shown as means ± SD (*n* = 3). ^##^ *p* < 0.01 vs. non-SiONPs-induced NCI-H292 cells, ^*^,^**^ *p* < 0.05 and < 0.01 vs. SiONPs-induced NCI-H292 cells, respectively.

As compared to the control group, the mRNA expression of TNF-α, IL-6, and IL-1β was increased in the SiONPs-treated cells (Figure 1a, Figure 1b, and Figure 1c, respectively). In contrast, compared to the SiONPs-only treated cells, silibinin significantly inhibited the elevation of the SiONPs-induced mRNA expression of proinflammatory cytokines in a concentration-dependent manner.

### 3.2. Effect of Silibinin on the Expression of TXNIP, MAPK, and AP-1 in NCI-H292 Cells Stimulated with SiONPs

**Figure 2:**
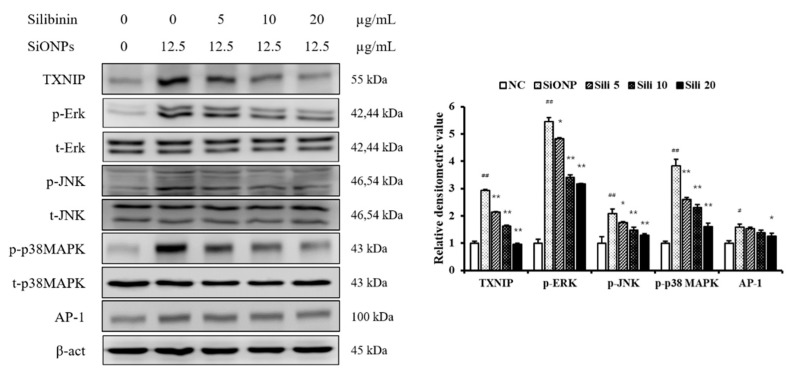
Effects of silibinin treatment on thioredoxin-interacting protein (TXNIP), mitogen-activated protein kinases (MAPK), and activator protein-1 (AP-1) expressions in SiONPs-induced NCI-H292 cells. Protein expression was determined by Western blotting analysis, and its densitometric value was assessed. Data are shown as means ± SD (*n* = 3). ^##^ *p* < 0.01 vs. non-SiONPs-induced NCI-H292 cells, * *p* < 0.05 and ** *p* < 0.01 vs. SiONPs-induced NCI-H292 cells, respectively.

At basal condition, Silibinin treatment decreased the TXNIP expression in NCI-H292 cells (Supplementary Figure S2). However, SiONPs treatment increased the TXNIP expression in NCI-H292 cells at basal condition (Supplementary Figure S3). SiONPs-stimulated cells significantly increased TXNIP expression compared to the control (Figure 2). However, silibinin treatment reduced TXNIP expression in SiONPs-stimulated cells. The phosphorylation of ERK, JNK, and p-38, increased in SiONPs-stimulated cells compared to the control; however, this phosphorylation was reduced by silibinin treatment, consistent with the results of TXNIP expression. In addition, silibinin treatment suppressed AP-1 expression induced by SiONPs stimulation.

**Figure 3:**
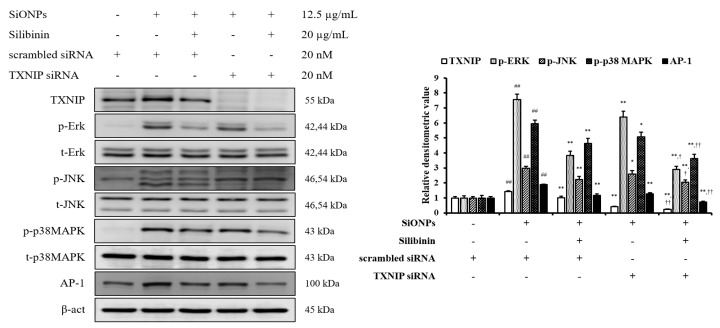
Effects of TXNIP in the effects of silibinin on SiONPs-induced NCI-H292 cells. Protein expression was determined by Western blotting analysis, and its densitometric value was assessed. Data are shown as means ± SD (*n* = 3). ^##^ *p* < 0.01 vs. non-SiONPs-induced NCI-H292 cells, ^*^,^**^ *p* < 0.05 and < 0.01 vs. SiONPs-induced NCI-H292 cells, respectively, ^†^,^††^ *p* < 0.05 and < 0.01 vs. silibinin-treated NCI-H292 cells induced by SiONPs, respectively.

In addition, we had performed an in vitro experiment using siRNA to investigate the role of TXNIP in the effects of silibinin on SiONPs-stimulated cells. The control siRNA did not affect the TXNIP and MAPKs expressions, whereas TXNIP siRNA reduced their expressions (Figure 3). Furthermore, silibinin and TXNIP siRNA treated cells with SiONPs application showed a more decreased TXNIP and MAPKs expression than the silibinin and SiONPs-treated cells.

### 3.3. Effects of Silibinin on the Recruitment of Inflammatory Cell in the BALF from SiONPs-Treated Mice

**Figure 4:**
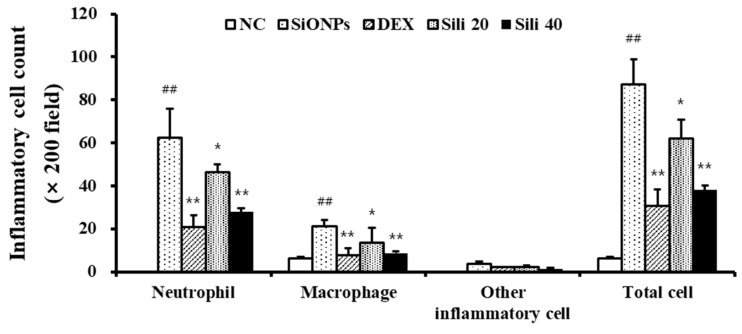
Inflammatory cell counts of the bronchoalveolar lavage fluid (BALF) from mice exposed to SiONPs. NC, normal control mice treated with PBS only; SiONPs, mice treated with SiONPs intranasal instillation; DEX, mice treated with dexamethasone (2 mg/kg) + SiONPs intranasal instillation; Sili 20 and 40, mice treated with silibinin (20 and 40 mg/kg, respectively) + SiONPs intranasal instillation. Data are represented as means ± SD (*n* = 6). ^##^ *p* < 0.01 vs. NC group, ^*^,^**^ *p* < 0.05 and < 0.01 vs. SiONPs group, respectively.

Compared to normal controls, SiONPs-treated mice showed increased total cell counts, particularly of neutrophils and macrophages, in their BALF (Figure 4). DEX-treated mice showed a reduction in inflammatory cell count, including macrophages and neutrophils, compared to SiONPs-treated mice. Similarly, there was a significant decline in the inflammatory cell count in silibinin-treated mice compared to SiONPs-treated mice; this decline was clearly detected in the group with 40 mg/kg silibinin.

### 3.4. Effects of Silibinin on Proinflammatory Mediators in the BALF from SiONPs-Treated Mice

**Figure 5:**
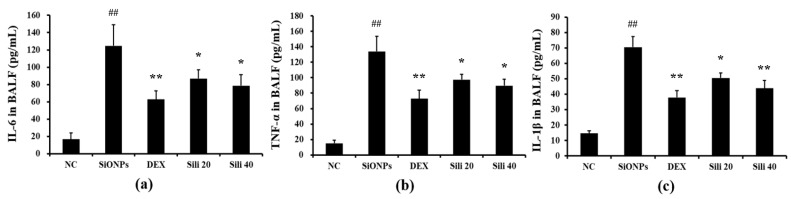
Effects of silibinin treatment on cytokines levels in BALF from mice exposed to SiONPs. (**a**) IL-6 level in BALF, (**b**) TNF-α level in BALF, (**c**) IL-1β level in BALF. NC, normal control mice treated with PBS only; SiONPs, mice treated with SiONPs intranasal instillation; DEX, mice treated with dexamethasone (2 mg/kg) + SiONPs intranasal instillation; Sili 20 and 40, mice treated with silibinin (20 and 40 mg/kg, respectively) + SiONPs intranasal instillation. Data are represented as means ± SD (*n* = 6). ^##^ *p* < 0.01 vs. NC group, ^*^,^**^ *p* < 0.05 and < 0.01 vs. SiONPs group, respectively.

Compared to normal controls, SiONPs-treated mice showed markedly elevated production of TNF-α and IL-6 in their BALF (Figure 5a and Figure 5b, respectively). However, DEX- and silibinin-treated groups showed significant decreases in the production of TNF-α and IL-6 compared to the levels in SiONPs-treated mice. In addition, the elevated IL-1β level in SiONPs-treated mice was reduced by silibinin administration, consistent with the results of TNF-α and IL-6 (Figure 5c).

### 3.5. Effects of Silibinin on Airway Inflammation in SiONPs-Treated Mice

**Figure 6:**
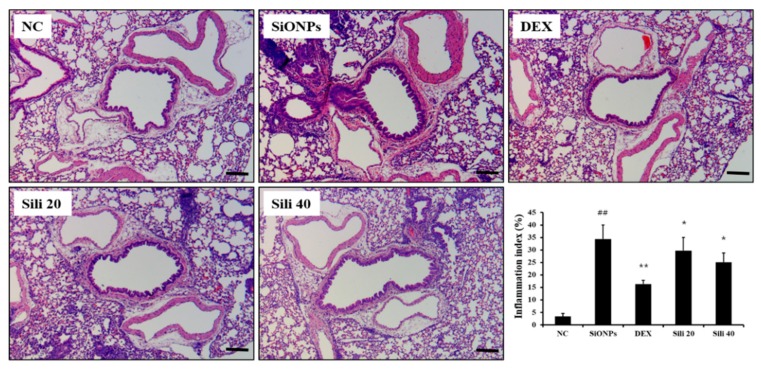
Effects of silibinin treatment on pulmonary inflammation caused by SiONPs exposure. A representative figure in the lung tissue stained with hematoxylin and eosin (×200). Quantitative analysis of inflammatory response was determined by an image analyzer. Scale bars indicate 50 μm. NC, normal control mice treated with PBS only; SiONPs, mice treated with SiONPs intranasal instillation; DEX, mice treated with dexamethasone (2 mg/kg) + SiONPs intranasal instillation; Sili 20 and 40, mice treated with silibinin (20 and 40 mg/kg, respectively) + SiONPs intranasal instillation. Data are represented as means ± SD (*n* = 6). ^##^ *p* < 0.01 vs. NC group, ^*^,^**^ *p* < 0.05 and < 0.01 vs. SiONPs group, respectively.

Compared to normal controls, SiONPs-treated mice showed marked inflammatory cell infiltration into lung tissues (Figure 6 and Supplementary Figure S4). However, inflammatory cell infiltration was significantly reduced in DEX-treated mice compared to SiONPs-treated mice. Silibinin-treated mice also showed declined inflammatory cell infiltration compared to SiONPs-treated mice; this decline was evident in the high dose group (40 mg/kg).

### 3.6. Effects of Silibinin on the Expression of TXNIP, MAPKs, and AP-1 in SiONPs-Treated Mice

**Figure 7:**
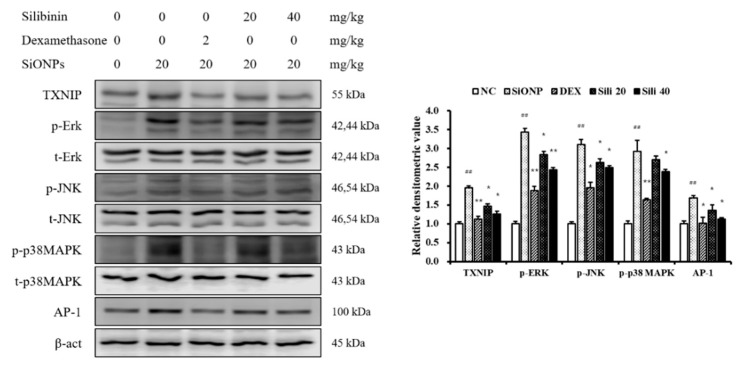
Effects of silibinin treatment on TXNIP, MAPK, and AP-1 expressions in the lung tissue from SiONPs-exposed mice. Protein expression was evaluated by Western blotting analysis, and densitometric value of protein expression was determined by Chemi-Doc. NC, normal control mice treated with PBS only; SiONPs, mice treated with SiONPs intranasal instillation; DEX, mice treated with dexamethasone (2 mg/kg) + SiONPs intranasal instillation; Sili 20 and 40, mice treated with silibinin (20 and 40 mg/kg, respectively) + SiONPs intranasal instillation. Data are represented as means ± SD (*n* = 6). ^##^ *p* < 0.01 vs. NC group, ^*^,^**^ *p* < 0.05 and < 0.01 vs. SiONPs group, respectively.

SiONPs-treated mice showed a marked elevation of TXNIP expression compared to the NC mice (Figure 7). However, DEX- and silibinin-treated mice showed a marked reduction in TXNIP expression compared to the SiONPs-treated mice. In addition, in comparison to the NC mice, SiONPs-treated mice showed marked elevations of the phosphorylation of ERK, JNK, and p38, as well as in the expression of AP-1. However, these increases induced by SiONPs exposure were significantly decreased by silibinin treatment, similar to the results of TXNIP expression.

### 3.7. Effects of Silibinin on TXNIP and AP-1 Expression in the Lung Tissues from SiONPs-Treated Mice

**Figure 8:**
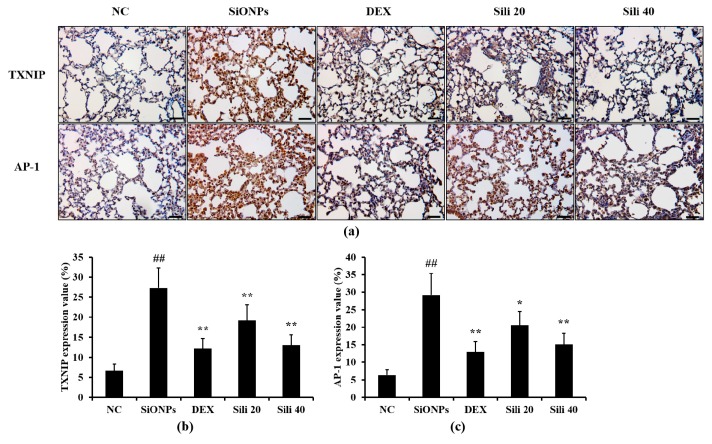
Effects of silibinin treatment on TXNIP and AP-1 expression in the lung tissue of SiONPs-induced mice. (**a**) A representative figure of an alveolar lesion in the lung tissue (×200), (**b**) TXNIP expression value, (**c**) AP-1 expression value. NC, normal control mice treated with PBS only; SiONPs, mice treated with SiONPs intranasal instillation; DEX, mice treated with dexamethasone (2 mg/kg) + SiONPs intranasal instillation; Sili 20 and 40, mice treated with silibinin (20 and 40 mg/kg, respectively) + SiONPs intranasal instillation. Scale bars = 50 μm. Data are represented as means ± SD (*n* = 6). ^##^ *p* < 0.01 vs. NC group, ^*^,^**^ *p* < 0.05 and < 0.01 vs. SiONPs group, respectively.

The SiONPs-treated mice showed increased TXNIP and AP-1 expression in their lung tissues compared to normal controls (Figure 8). However, compared to SiONPs-treated mice, DEX-treated mice showed decreased TXNIP and AP-1 expression in their lung tissues. Similarly, compared to SiONPs-treated mice, silibinin-treated mice showed reduced TXNIP and AP-1 expression in their lung tissues; this reduction was evident in the group with 40 mg/kg silibinin.

## 4. Discussion

Along with the rapid development of nanotechnology, concerns regarding the potential adverse effects of nanomaterials are increasing. Among the various nanomaterials, SiONPs induce pathophysiological alterations of various organs and, in particular, cause lung damage via excessive inflammatory responses [27,28]. In the present study, we explored the effects of silibinin on airway inflammation induced by SiONPs exposure in vivo and in vitro, with a focus on the modulation of TXNIP/MAPK/AP-1 signaling. Silibinin treatment reduced the elevations of proinflammatory mediators caused by SiONPs exposure in in vivo and in vitro and suppressed SiONPs-induced upregulation of TXNIP/MAPKs/AP-1 signaling.

It is known that exposure to SiONPs causes inflammatory responses in the respiratory tract via the elevation of inflammatory mediator production [27,28,29]. Particularly, inflammatory cytokines act as a key player in the recruitment of inflammatory cells and the activation of their migration into tissues [30,31]. Previous studies have shown that SiONPs exposure increases the production of TNF-α, IL-6, and IL-1β and elevates inflammatory cell recruitment into lung tissues, resulting in the aggravation of airway inflammation [8,32]. Therefore, reducing the levels of proinflammatory mediators is important for developing therapeutic agents to treat airway inflammation induced by SiONPs exposure. In this study, treatment with silibinin led to a marked reduction in the elevations of proinflammatory mediators in SiONPs-stimulated H292 cells. In addition, silibinin administration reduced inflammatory cell counts and cytokines in SiONPs-treated mice and also inhibited inflammatory cell infiltration into lung tissues. These results indicated that silibinin suppressed airway inflammation caused by SiONPs exposure. These effects caused by silibinin treatment were supported by previous studies [33,34], wherein silibinin alleviated inflammatory response in radiation- or methotrexate-induced pulmonary injury via suppression of cytokine production.

It is well known that MAPKs are involved in the development and aggravation of inflammatory responses, and they are grouped into three families, mainly ERK1/2, JNK, and p38 MAPK [35,36]. These MAPKs are phosphorylated by various stimuli and then activate AP-1 expression, resulting in the elevation of proinflammatory mediator production [37,38]. According to a previous study, MAPKs are closely related to SiONPs-induced inflammation [39]. SiONPs exposure induces the phosphorylation of MAPKs, which eventually causes the development and aggravation of airway inflammation. In this study, treatment with silibinin significantly reduced the phosphorylation of ERK1/2, JNK, and p38 in SiONPs-stimulated H292 cells and also reduced the expression of AP-1. These effects of silibinin were consistent with the result of our in vivo experiment. Silibinin administration markedly declined the phosphorylation of MAPKs and the expression of AP-1 in SiONPs-treated mice. These results were also supported by previous studies [24,25,40], in which silibinin attenuated cigarette smoke-induced pulmonary inflammation by suppressing ERK1/2 and downregulated the expression of MAPK and AP-1 in human lung carcinoma cells. Therefore, these results indicated that the therapeutic effects of silibinin on SiONPs-induced airway inflammation was involved in the suppression of MAPKs phosphorylation. In addition, treatment with silibinin significantly reduced TXNIP expression in SiONPs-stimulated H292 cells and SiONPs-treated mice. According to recent studies, TXNIP is involved in ROS production and MAPKs phosphorylation, and its overexpression induces the elevation of MAPKs phosphorylation, which, in turn, results in apoptosis and inflammation [19,41]. Therefore, TXNIP is considered an important factor in SiONPs-induced inflammatory responses. The suppression of TXNIP expression by silibinin was also supported by the results of our immunohistochemistry (IHC) examination. SiONPs-treated mice showed increased TXNIP expression on their lung tissues, whereas decreased TXNIP expression was observed in silibinin-treated mice compared to SiONPs-treated mice. These results indicated that silibinin was associated with the suppression of TXNIP/MAPKs/AP-1 signaling to alleviate SiONPs-induced airway inflammation.

## 5. Conclusions

Silibinin significantly declined the infiltration of inflammatory cells and the production of inflammatory mediators in human airway epithelial cells and lung tissues exposed to SiONPs, which were closely involved in the suppression of TXNIP/MAPKs/AP-1 signaling. Therefore, our study suggested that silibinin may be effective for the treatment of airway inflammation caused by SiONPs. In addition, further studies are needed to investigate the effects of silibinin on pulmonary inflammation caused by fine dust exposure as well as SiONPs in human to increase the potential as a therapeutic agent for silibinin.

## Supplementary Materials

The following are available online at https://www.mdpi.com/2073-4409/9/3/678/s1, Figure S1: Cell viability in the presence of silibinin in NCI-H292 cells, Figure S2: The effects of silibinin on TXNIP expression at the basal condition, Figure S3: The effects of SiONPs on TXNIP expression at the basal condition, Figure S4: Histopathology for lung tissue (magnification x100).
