# Supplementary material for: Silibinin Attenuates Silica Dioxide Nanoparticles-Induced Inflammation by Suppressing TXNIP/MAPKs/AP-1 Signaling
Source: Cells. 2020 Mar 10;9(3):678. doi: 10.3390/cells9030678 (PMC7140632; doi:10.3390/cells9030678)
Supplement: Supplementary file 1 [file cells-09-00678-s001.pdf]

Supplementary Materials

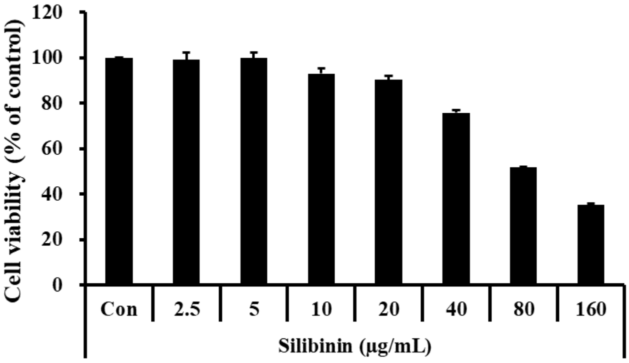

Supplementary figure 1. Cell viability in the presence of silibinin in NCI-H292 cells. Cell viability was measure using 3-(4,5-dimethylthiazol-2-yl)-2,5-dihenyltetrazolium bromide (MTT) assay.

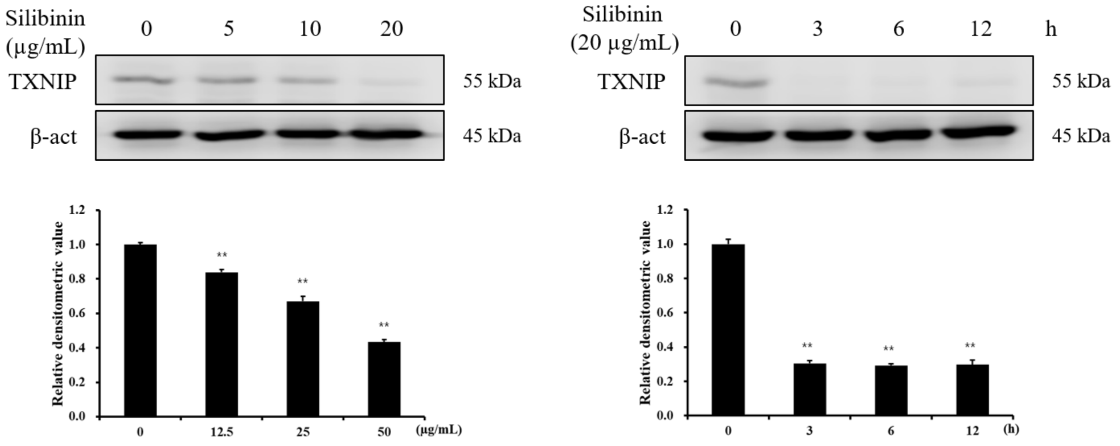

(a) **(b)**

Supplementary figure 2. The effects of silibinin on TXNIP expression at the basal condition (a) concentration dependent effect, (b) time dependent effect. ** $P < 0.01$ vs non-treated NCI-H292 cells.

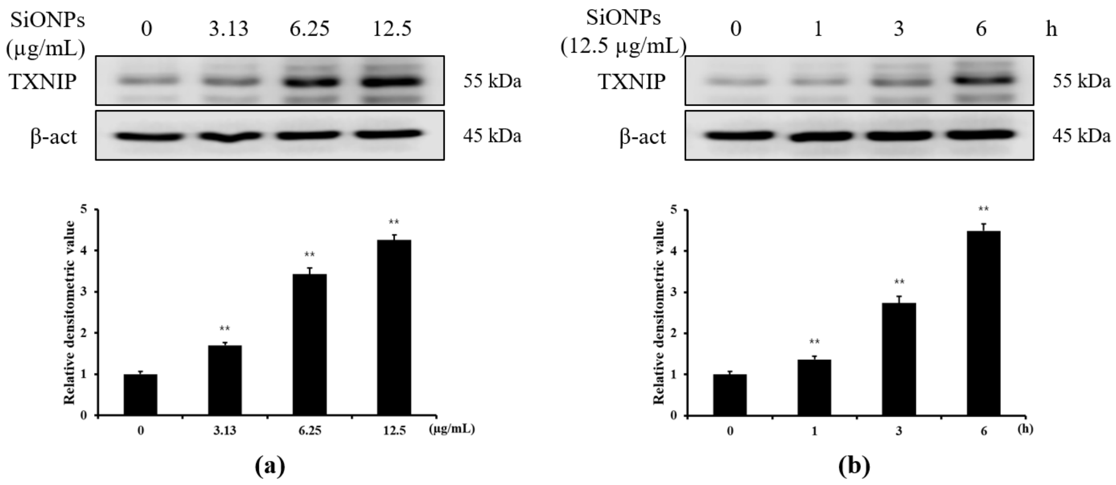

Supplementary figure 3. The effects of SiONPs on TXNIP expression at the basal condition (a) concentration dependent effect, (b) time dependent effect. ** $P < 0.01$ vs non-treated NCI-H292 cells.

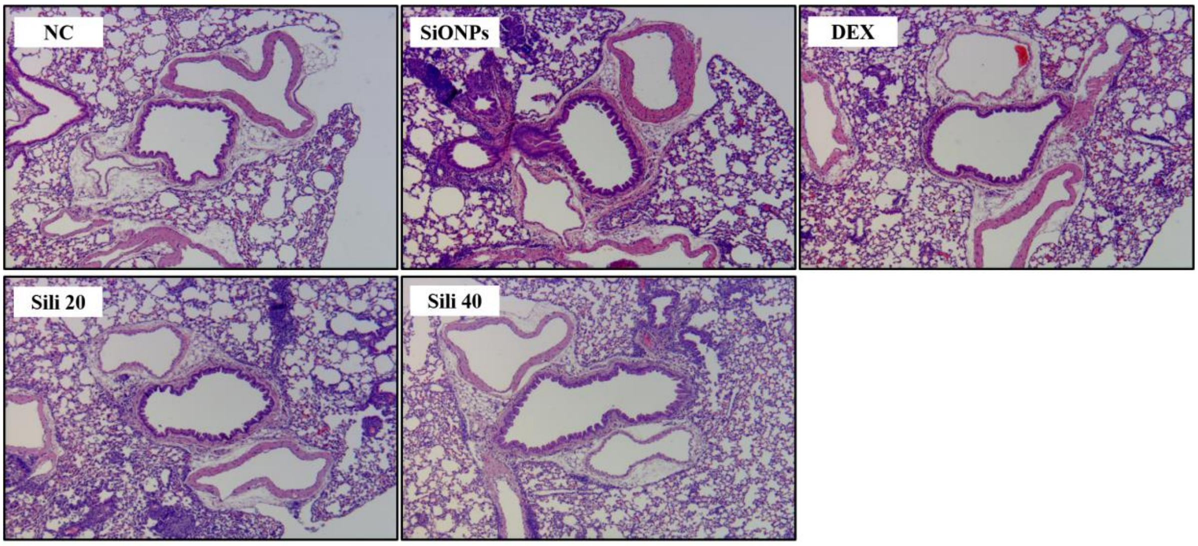

Supplementary figure 4. Histopathology for lung tissue (magnification x100).
